# Assessment of River Water Quality Based on an Improved Fuzzy Matter-Element Model

**DOI:** 10.3390/ijerph16152793

**Published:** 2019-08-05

**Authors:** Yumin Wang, Weijian Ran, Lei Wu, Yifeng Wu

**Affiliations:** 1 School of Energy and Environment, Southeast University, Nanjing 210096, China; 2 School of Glasgow, University of Electronic Science and Technology, Chengdu 610054, China

**Keywords:** improved fuzzy matter-element model (IFME), set pair analysis (SPA), variation coefficient method (VCM), water quality assessment

## Abstract

In this paper, an improved fuzzy matter-element (IFME) method was proposed, which integrates the classical matter-element (ME) method, set pair analysis (SPA), and variable coefficient method (VCM). The method was applied to evaluate water quality of five monitor stations along Caoqiao River in Yixing city, Jiangsu Province, China. The levels of river water quality were determined according to fuzzy closeness degree. Compared with the traditional evaluation methods, the IFME method has several characteristics as follows: (i) weights were determined by the VCM method, which can reduce workload and overcome the adverse effects of abnormal values, (ii) membership degrees were defined by SPA, which can utilize monitored data more scientifically and comprehensively, and (iii) IFME is more suitable for seriously polluted rivers. Overall, these findings reinforce the notion that an integrated approach is essential for attaining scientific and objective assessment of river water quality.

## 1. Introduction

River water pollution is one of the most widespread environmental issues in the 21st century [1,2,3]. Since rivers carry off domestic sewage, industrial wastewater, and agricultural discharge, as well as serve as vital water sources for populations, irrigation, industry, and other applications, it plays significant roles in the economic development of watersheds [4,5]. To analyze causes of water pollution and formulate river management policies, it is necessary to assess river water quality scientifically and objectively [6,7,8].

Nowadays, a rising number of mathematical approaches have been extensively developed to evaluate water quality, such as multivariate statistical analysis (MVSA) [4,9,10,11,12,13,14], artificial neural networks [15,16], fuzzy comprehensive assessments [17,18], matter-element analysis [6], etc. These mathematical assessments methods have been widely applied for researchers to help solve problems in water-related environmental management. However, all the methods mentioned above have their own limitations. In the application of MVSA, large amounts of samples are required, and artificial neural networks lacks accurate analysis of each performance index. Therefore, improving the disadvantages of each method and combining the advantages of various methods are indispensable in comprehensive assessments [9,19].

Matter-element theory was first introduced for solving incompatible problems qualitatively and quantitatively in the 1980s by the Chinese mathematician Cai Wen [20], which has been widely used in differing fields including pattern recognition, scientific decision and comprehensive evaluation. In addition, set pair analysis (SPA), proposed by Keqin Zhao in 1989, is a modified theory about uncertainty, and have been applied to fields of multi-objective decision making and multi-attribute assessment communities related to management, applied math, computer science, social science, environmental assessment, and management [21]. SPA can deal with issues with dialectic characteristics by integrating both certainties and uncertainties into certain–uncertain system and depicting the certainty and uncertainty systematically from three aspects of identity, discrepancy, and contrary. The variation coefficient method (VCM) can be applied to determine the weights of indicators, which reflect the variation of objective information as well as reduces the workload. In this paper, SPA was embedded into matter-element analysis to build an improved fuzzy matter-element (IFME) model to assess the river water quality in a scientific and objective way with the weights determined by VCM.

In this paper, firstly, IFME was proposed based on the fuzzy matter-element (FME) model, and detailed procedures on how to perform the IFME model was illustrated. Secondly, the method was employed to evaluate river water quality and compared with the other methods. Finally, a specific river was taken as an example to evaluate the water quality, and discussions and conclusions about the river water quality assessment were made.

## 2. Methodology

### 2.1. Study Area

**Figure 1:**
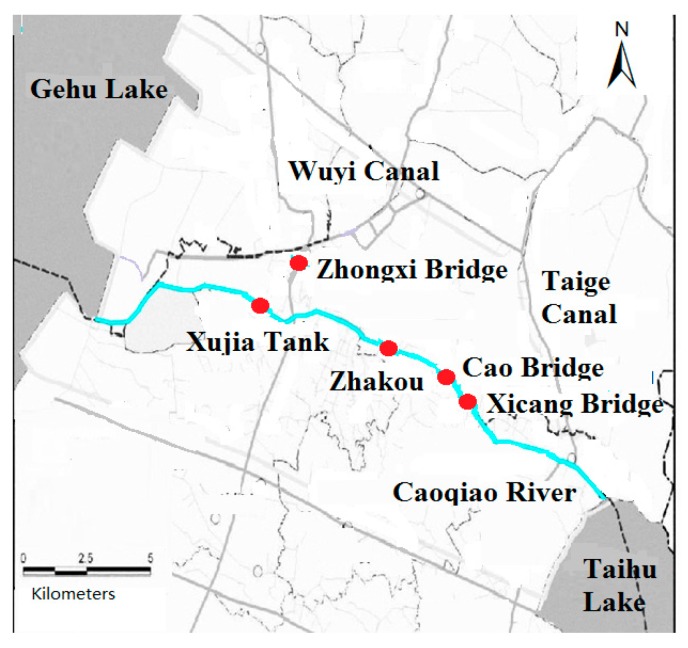
Map of the study area with locations of water quality monitoring stations.

Caoqiao River is located in the south of Jiangsu Province in China with a total length of 21.5 km. The upstream and downstream of the Caoqiao River intersects with the Wuyi canal and Taige canals, respectively. Caoqiao River converges into Taihu Lake at Baidu port. Caoqiao River is seriously polluted due to over-developed industry and dense population distribution along the river, which leads to water quality even reaching Grade “V” in the Chinese environmental quality standards for surface water (GB3838-2002) [22], shown in Table 1. The water quality data at the five monitoring stations of Zhongxi Bridge, Xujia Tank, Zhakou, Cao Bridge and Xicang Bridge were collected from the Yixing Environmental Monitor Station, shown in Figure 1. The water quality parameters analyzed include the permanganate index (COD_Mn_), chemical oxygen demand (COD_Cr_), ammonia nitrogen (NH_3_-N), total phosphorus (TP), and total nitrogen (TN).

### 2.2. Improved Fuzzy Matter-Element (IFME) Model

A basic matter-element $R_{mn}$ is established by *m* assessment objects (stations), *n* feature vectors (indicators), and the corresponding characteristic values *x_mn_* (indicator values), expressed in the matrix format by Equation (1) as follows:

(1) $$R_{mn}=\left[\begin{array}{cccccc} x_{11} & x_{12} & \cdots & x_{1j} & \cdots & x_{1n}\\ x_{21} & x_{22} & \cdots & x_{2j} & \cdots & x_{2n}\\ \vdots & \vdots & \vdots & \vdots & \vdots & \vdots \\ x_{i1} & x_{i2} & \cdots & x_{ij} & \cdots & x_{in}\\ \vdots & \vdots & \vdots & \vdots & \vdots & \vdots \\ x_{m1} & x_{m2} & \cdots & x_{mj} & \cdots & x_{mn} \end{array}\right].$$

In the fuzzy matter element model, characteristic values were expressed by triangular, trapezoidal, and normal distribution functions of indicators, without sufficient consideration of the monitored data. In this paper, the SPA method is applied to determine membership degrees, which considers the relationship between indicators and water quality grades more objectively and comprehensively.

#### 2.2.1. Determination of Membership Degree in the Fuzzy Matter Element Model by SPA

A set pair is formed by putting two interrelated sets (matter-elements) together to determine the membership degree of indicators. The connection degree of the two sets, including identity degree, discrepancy degree and contrary degree under certain circumstances is given by Equation (2) as follows:

(2) $$\mu =\frac{S}{N}+\frac{F}{N}i+\frac{P}{N}j$$

where μ is the connection degree of the set pair, *N* denotes the total number of characteristics of the set pair, *S* represents the number of identity characteristics, *P* is the number of contrary characteristics, F=N−S−P is the number of the characteristics of these two sets that are neither identity nor contrary, $\frac{S}{N}$**,** $\frac{F}{N}$**,** and $\frac{P}{N}$ represents identity degree, discrepancy degree and contrary degree, respectively, *j* is the coefficient of the contrary degree, and is specified as −1, and *i* is an uncertain value between −1 and 1, i.e., i∈[−1,1], in terms of various circumstances. The uncertainty of the discrepancy degree of two sets is eliminated when *i* is specified as −1 or 1, and will increase when *i* is approaching zero.

Given *a* = *S*/*N*, *b* = *F*/*N*, *c* = *P*/*N*, Equation (2) can be rewritten by Equation (3) as follows:

(3) μ=a+bi+cj.

#### 2.2.2. Determination of Weights by VCM

The weight of index has direct and significant influence on the assessment results now that different weights will lead to diverse results. Therefore, the effectivity of the river water quality assessment is determined by the index weight to a certain extent [21]. In the evaluation system, an indicator with a greater variation coefficient means that it can provide more information, which should be given a higher weight. The method to determine weights of indicators is termed the variation coefficient method (VCM), which was described as follows:

1. Normalize the indicator value matrix.
  The normalized matrix $R^{\prime }={\left(x_{ij}^{\prime }\right)}_{mn}$ is obtained by Equation (4-1) as follows:
  (4-1) $$x_{ij}^{\prime }=\{\begin{array}{cc} \frac{x_{ij}-\min\{x_{ij}\}}{\max\{x_{ij}\}-\min\{x_{ij}\}}, & x_{ij}\;\mathrm{is}\;a\;\mathrm{efficiency}\;\mathrm{indicator}\\ \frac{\max\{x_{ij}\}-x_{ij}}{\max\{x_{ij}\}-\min\{x_{ij}\}}, & x_{ij}\;\mathrm{is}\;a\;\cos t\;\mathrm{indicator} \end{array}$$
  where efficiency indicators refer to indicators that correlate positively with normalization results, and cost indicators refer to indicators that correlate negatively with normalization results.
2. Calculate the average value of each indicator $\bar{x_{j}}$:
  (4-2) $$\bar{x_{j}}=(\sum_{i=1}^{m}x_{{}_{ij}}^{\prime })/m.$$
3. Calculate the mean squared deviation of each indicator $D_{j}$:
  (4-3) $$D_{j}=\sqrt{\frac{\sum_{i=1}^{m}{\left(x_{{}_{ij}}^{\prime }-\bar{x_{j}}\right)}^{2}}{m-1}}.$$
4. Calculate the variable coefficient of each indicator $C_{Vj}$:
  (4-4) $$C_{Vj}=\frac{D_{j}}{\bar{x_{j}}}.$$
5. Determine the weight of each indicator $w_{j}$ by normalization:
  (4-5) $$w_{j}=\frac{C_{Vj}}{\sum_{j=1}^{n}C_{Vj}}.$$

### 2.3. River Water Quality Assessment by the IFME Method

#### 2.3.1. Selection of Water Quality Indicators

In the environmental quality standards for surface water (GB3838-2002) [22], which is the national standard of the People’s Republic of China, water quality is classified into five levels, shown in Table 1, and the connection degree can be calculated by the SPA method.

The evaluation standard matter-element matrix *S_nk_* is expressed by Equation (5) as follows:

(5) $$S_{nK}=\left[\begin{array}{cccccc} s_{11} & s_{12} & \cdots & s_{1k} & \cdots & s_{1K}\\ s_{21} & s_{22} & \cdots & s_{2k} & \cdots & s_{2K}\\ \vdots & \vdots & \vdots & \vdots & \vdots & \vdots \\ s_{j1} & s_{j2} & \cdots & s_{jk} & \cdots & s_{jk}\\ \vdots & \vdots & \vdots & \vdots & \vdots & \vdots \\ s_{n1} & s_{n2} & \cdots & s_{nk} & \cdots & s_{nK} \end{array}\right]$$

where $s_{jk}$ is the upper boundary value of the *j*th indicator in the *k*th grade, and the lower boundary $s_{j0}$ is equal to 0 (j=1,2,⋯,n).

#### 2.3.2. Calculation of the Membership Degree of Each Indicator

In general, if $x_{ij}\in [s_{j,k-1},s_{jk}]$, then the connection degree $\mu_{jk}$ is equal to 1; if $x_{ij}\in [s_{j,k-3},s_{j,k-2}]$ or $x_{ij}\in [s_{j,k+1},s_{j,k+2}]$, then the connection degree $\mu_{jk}$ is equal to −1; if $x_{ij}\in [s_{j,k-2},s_{j,k-1}]$ or $x_{ij}\in [s_{jk},s_{j,k+1}]$, then the connection degree $\mu_{jk}$ is defined by Equation (6) as follows:

(6) $$\mu_{jk}=\{\begin{array}{cc} 1-\frac{2(s_{j,k-1}-x_{ij})}{s_{j,k-1}-s_{j,k-2}}, & x_{ij}\in [s_{j,k-2},s_{j,k-1}]\\ 1-\frac{2(x_{ij}-s_{jk})}{s_{j,k+1}-s_{jk}}, & x_{ij}\in [s_{jk},s_{j,k+1}] \end{array}.$$

Then the connection degree matter element matrix is expressed by Equation (7) as follows:

(7) $$\mu_{nK}^{m}=\left[\begin{array}{cccccc} \mu_{11} & \mu_{12} & \cdots & \mu_{1k} & \cdots & \mu_{1K}\\ \mu_{21} & \mu_{22} & \cdots & \mu_{2k} & \cdots & \mu_{2K}\\ \vdots & \vdots & \vdots & \vdots & \vdots & \vdots \\ \mu_{j1} & \mu_{j2} & \cdots & \mu_{jk} & \cdots & \mu_{jK}\\ \vdots & \vdots & \vdots & \vdots & \vdots & \vdots \\ \mu_{n1} & \mu_{n2} & \cdots & \mu_{nk} & \cdots & \mu_{nK} \end{array}\right]$$

where *n* is the number of assessment indicators and *K* is the number of assessment grades. In addition, if $x_{ij}>s_{j5}$, then the connection degree $\mu_{j6}=1$, meanwhile $\mu_{j5}=-1$.

The membership degree $v_{jk}$ can be calculated by Equation (8) as follows:

(8) $$v_{jk}=(1+\mu_{jk})/2.$$

#### 2.3.3. Generation of the Compound Fuzzy Matter-Element

The sum of each line should be equal to 1 due to each element in this line being the membership function of each grade. Therefore, each element of each line in this matrix is normalized by Equation (9) as follows:

(9) $$r_{jk}=\frac{v_{jk}}{\sum_{k=1}^{K}v_{jk}}.$$

Based on the normalization, the compound fuzzy matter-element matrix of the *i*th assessment object is generated by Equation (10) as follows:

(10) $$r_{nK}^{m}=\left[\begin{array}{cccccc} r_{11} & r_{12} & \cdots & r_{1k} & \cdots & r_{1K}\\ r_{21} & r_{22} & \cdots & r_{2k} & \cdots & r_{2K}\\ \vdots & \vdots & \vdots & \vdots & \vdots & \vdots \\ r_{j1} & r_{j2} & \cdots & r_{jk} & \cdots & r_{jK}\\ \vdots & \vdots & \vdots & \vdots & \vdots & \vdots \\ r_{n1} & r_{n2} & \cdots & r_{nk} & \cdots & r_{nK} \end{array}\right].$$

#### 2.3.4. Calculation of Fuzzy Closeness Degree

The fuzzy closeness degree is defined as a measure that shows the proximity between the evaluated and standard matter-element in similar fashion to a similarity coefficient in multivariate statistical analysis. A greater value of fuzzy closeness degree indicates a smaller proximity. Thus, all evaluated samples can be sorted according to their fuzzy closeness degree, and can also be classified according to the fuzzy closeness degree of standard samples. In this paper, the ideal matrix of the normalized fuzzy matter-element is:

(11) $$R_{0K}=\left|\begin{array}{c} r_{01}\\ r_{02}\\ \vdots \\ r_{0k}\\ \vdots \\ r_{0K} \end{array}\right|=\left|\begin{array}{c} 1\\ 1\\ \vdots \\ 1\\ \vdots \\ 1 \end{array}\right|.$$

Then, the fuzzy closeness degree matrix of the *m*th assessment object is generated by using the Hamming closeness degree (ρH) and the weighted average fuzzy arithmetic operator (·, +), as follows:

(12) $$\rho H_{mK}=\left|\begin{array}{cccccc} \rho H_{11} & \rho H_{12} & \cdots & \rho H_{1k} & \cdots & \rho H_{1K}\\ \rho H_{21} & \rho H_{22} & \cdots & \rho H_{2k} & \cdots & \rho H_{2K}\\ \vdots & \vdots & \vdots & \vdots & \vdots & \vdots \\ \rho H_{i1} & \rho H_{i2} & \cdots & \rho H_{ik} & \cdots & \rho H_{iK}\\ \vdots & \vdots & \vdots & \vdots & \vdots & \vdots \\ \rho H_{m1} & \rho H_{m2} & \cdots & \rho H_{mk} & \cdots & \rho H_{mK} \end{array}\right|,$$

(13) $$\rho H_{ik}=1-\sum_{j=1}^{n}w_{j}\cdot \left|r_{jk}-r_{0k}\right|,i=1,2,\ldots ,m.$$

#### 2.3.5. Calculation of the Grade Characteristic Value (H) and Grade of the River Water Quality

The grade characteristic value of the *i*th assessment object (*H_i_*) is calculated by Equation (14) as follows:

(14) $$H_{i}=\sum_{k=1}^{K}\frac{k\rho H_{ik}}{\sum_{k=1}^{K}\rho H_{ik}}$$

where the water quality grade of the *i*th assessment object is “VI” when 5.5 < *H_i_* < 6, which means that water quality is inferior to level V; the water quality grade of the *i*th assessment object is “I” when 1 < *H_i_* ≤ 1.5; the water quality grade of the *i*th assessment object is X when $\begin{array}{cc} X-0.5<H_{i}\leq X+0.5, & X=2,3,4,5 \end{array}$.

## 3. Results and Discussion

### 3.1. Water Quality Assessment Results of Caoqiao River

**Figure 2:**
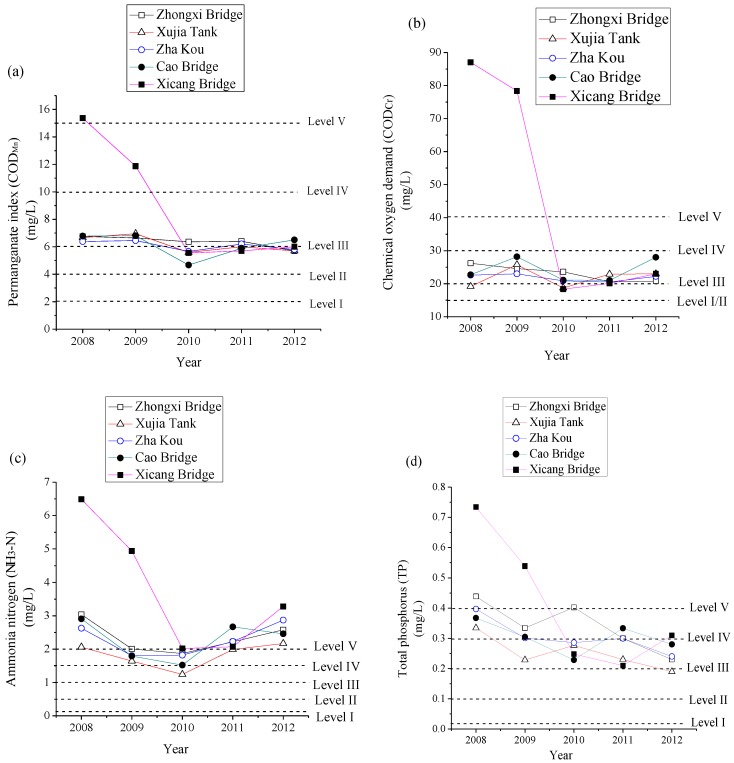

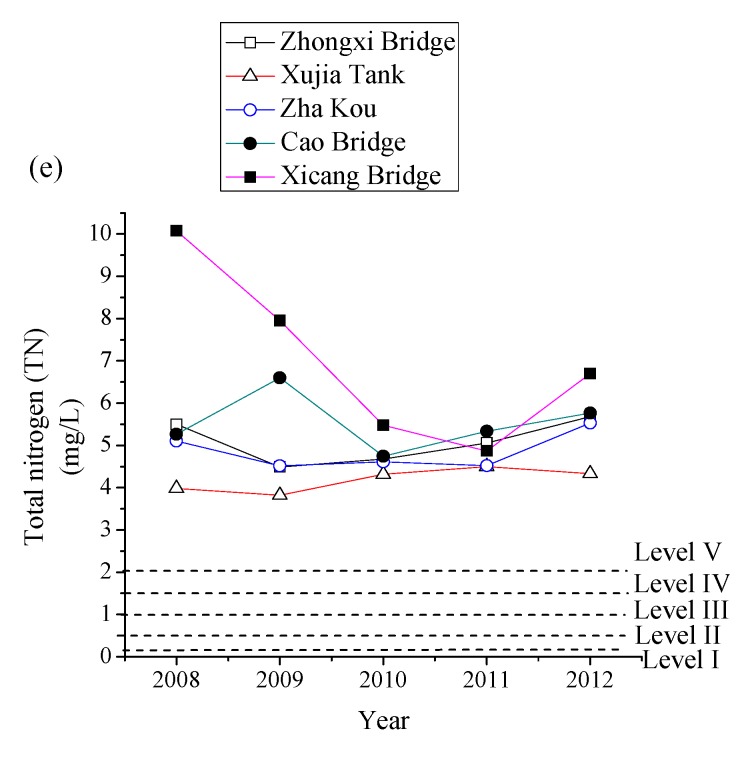
Yearly water quality of five monitoring stations in Caoqiao River. (**a**) Permanganate index, (**b**) Chemical oxygen demand, (**c**) Ammonia-nitrogen, (**d**) Total phosphorus, and (**e**) Total nitrogen.

The water quality data of the indicators for the five monitoring stations are shown in Figure 2.

It can be observed from Figure 2 that the values of water quality indicators for Xicang Bridge are the highest among all five monitoring stations, while the values of water quality indicators for Xujia Tank are the lowest among all five monitoring stations. Generally speaking, for most of the monitoring stations, the values of water quality indicators in 2010 are the lowest during the period from 2008 to 2012, which means that the water quality was the best in 2010.

The IFME model was established based on water quality data from five monitoring stations along the Caoqiao River. The membership function of each indicator was determined by SPA expressed by Equations (2) and (3). The weight of each indicator was calculated by VCM expressed by Equation (4-1)–(4-5), and shown in Table 1. The fuzzy matter-element matrix of water quality assessment was established by Equations (5) and (10) based on the membership function of indicators. The fuzzy closeness degree and grade characteristic value for each section in 2010 was computed by Equations (11) and (14), and shown in Table 2.

The grade characteristic values of five monitoring sections of Caoqiao River in 2010 decreased in the order of Zhongxi Bridge (5.04) > Zhakou (4.51) > Xicang Bridge (4.45) > Xujia Tank (4.23) > Cao Bridge (4.20). According to the classification rule of the grade characteristic value, the river water quality of the five stations ranges from level “IV” to level “V”, which are “V”, “IV”, “V”, “IV”, and “IV”, respectively. The results indicated that the water quality level of Zhongxi Bridge station is more seriously affected than the other four stations [23]. Since Zhongxi Bridge station is located in the Wuyi Canal, which is upstream of Caoqiao River, about 2554 tons of COD_cr_, 314.6 tons of NH_3_-N, 506 tons of TN, and 49.5 tons of TP were brought into the Caoqiao river, which leads to the deterioration of water quality [24]. Although the water quality seems to be improved from level “V” to level “IV”, the water quality in the studied river is still at a relatively low level and it still has great potential in the future, and it will remain urgent to improve the water quality through measures conducted by authorities and citizens.

The IFME model was applied to five monitoring stations from 2008 to 2012, and results are shown in Table 3.

The average grade characteristic values *H_m_* from 2008 to 2010 for five stations increased in the order of Xujia Tank (4.48) < Zhakou (4.69) < Cao Bridge (4.71) < Zhongxi Bridge (4.88) < Xicang Bridge (5.13), which indicated that the water quality level decreased in the order of Xujia Tank > Zhakou > Cao Bridge > Zhongxi Bridge > Xicang Bridge. Generally speaking, the grade characteristic value in 2010 is the lowest. Take Xujia Tank as an example, the grade characteristic values *H_m_* decreased in the order of 2008 (4.76) > 2011 (4.49) > 2009 (4.48) > 2012 (4.45) > 2010 (4.23), which indicated that water quality level in 2010 was better than other years. The trend of “first decrease rapidly, and afterwards rise slowly from year 2008 to year 2012” occurred for five stations. In addition, the grade characteristic values *H_m_* for the five monitoring stations in 2012 were all lower than that in 2008, which indicted that water quality in general is getting better from 2008 to 2012 along the Caoqiao River.

### 3.2. Compared with Other Different Methods

In order to validate the improved fuzzy matter-element model in this study, we employed four widely used comprehensive assessment methods to evaluate the river water quality of five stations along the Caoqiao River, shown in Table 4. The assessment results of the five stations using the fuzzy matter-element model as well as comprehensive index method are completely consistent with those using the improved fuzzy matter-element model. Meanwhile, the assessment results of the fuzzy comprehensive method and the Bayesian method are also identical. However, the assessment results of the fuzzy comprehensive method and the Bayesian method are not consistent with the assessment results of the other three methods in all stations except Zhongxi Bridge station.

In terms of the five grades, the membership functions of the fuzzy comprehensive assessment and the Bayesian method are shown in Table 5 and Table 6. However, it is unreasonable because it omitted much information of the other grades. When the water quality indicator is worse than Level V, the membership functions of both the fuzzy comprehensive assessment and the Bayesian method in Level V are defined as 1.0, which raised the evaluating degree due to the serious pollution of the Caoqiao river, with TN higher than the standard for Level “V”. Therefore, it is reasonable that the river water quality of all stations except Zhongxi Bridge is ranked Level “IV”. In conclusion, the assessment results of both the fuzzy comprehensive method and the Bayesian method should be consistent with the assessment results using the improved fuzzy matter-element model.

According to the abovementioned analysis, the improved fuzzy matter-element model has several advantages as follows: Firstly, this improved model can effectively solve the problem of water quality assessments of seriously polluted rivers when that water quality indicator is out of the range of criteria. When the grade characteristic value is greater than 5.5, the water quality is cataloged into level VI. The river is polluted more seriously with a greater grade characteristic value. Secondly, SPA considers the transformation tendency of water quality by identical degree, discrepancy degree, and contrary degree to define the membership degree, which can objectively reflect the total difference between the assessed matter-element and the standard matter-element comprehensively, to make the evaluation results more accurate and reasonable, and make use of data sufficiently and effectively. Furthermore, all information in each assessment grade can be taken into account in the comprehensive assessment. Last but not the least, the variable coefficient method considers the weight of each indicator under all evaluating objects as the same, so the workload is alleviated greatly, and it can determine weights of all indicators as objectively as possible. It gives a greater weight to the indicator which has a larger variation coefficient and carries more information, and this can distinguish the weights well and avoid equalization. Therefore, the improved fuzzy matter-element model can be applied to the comprehensive assessment of river water quality.

## 4. Conclusions

Developing comprehensive methods for water quality assessments for rivers is indispensable in river management. In this paper, an improved fuzzy matter-element (IFME) model was proposed and applied to assess river water quality of the Caoqiao River. The IFME model integrated fuzzy matter-element, SPA, and VCM, with membership degrees determined by SPA and weights defined by VCM. The results obtained indicated that the overall water quality level of the Caoqiao River is level “IV” or Level “V”. Particularly, the water quality level of Zhongxi Bridge is Level “V”. Compared with other methods, the IFME model assessed river water quality more effectively and feasibly. Therefore, it provides a useful reference for river water quality assessment in other seriously polluted rivers.

## Figures and Tables

**Table 1:** Upper boundary values of indicators in environmental quality standards for surface water (GB3838-2002) (unit: mg/L).

Indicators	I	II	III	IV	V	Weights
COD_Mn_	2	4	6	10	15	0.15
COD_Cr_	15	15	20	30	40	0.22
NH_3_-N	0.15	0.5	1	1.5	2	0.16
TP	0.02	0.1	0.2	0.3	0.4	0.25
TN	0.2	0.5	1	1.5	2	0.22

**Table 2:** Closeness degree and river water quality level in 2010 for five stations along the Caoqiao River.

Station	I	II	III	IV	V	Below Level V	*H_m_*	Level
Zhongxi Bridge	0.00	0.00	0.14	0.21	0.12	0.53	5.04	V
Xujia tank	0.00	0.04	0.26	0.35	0.14	0.22	4.23	IV
Zhakou	0.00	0.01	0.19	0.33	0.20	0.27	4.51	V
Cao Bridge	0.00	0.05	0.27	0.34	0.13	0.22	4.20	IV
Xicang Bridge	0.00	0.05	0.25	0.26	0.06	0.37	4.45	IV

**Table 3:** Grade characteristic values and river water quality level from 2008 to 2010 for five stations along the Caoqiao River.

Station	2008		2009		2010		2011		2012		Avarage
	*H_m_*	Level	*H_m_*	Level	*H_m_*	Level	*H_m_*	Level	*H_m_*	Level	*H_m_*
Zhongxi Bridge	5.24	V	4.90	V	5.04	V	4.71	V	4.50	V	4.88
Xujia Tank	4.76	V	4.48	IV	4.23	IV	4.49	IV	4.45	IV	4.48
Zhakou	5.01	V	4.63	V	4.51	V	4.71	V	4.57	V	4.69
Cao Bridge	4.95	V	4.77	V	4.20	IV	4.79	V	4.83	V	4.71
Xicang Bridge	6.00	VI	5.83	VI	4.45	IV	4.44	IV	4.94	V	5.13

**Table 4:** Comparison of the assessment results of different methods.

Methods	Zhongxi Bridge	Zhakou	Xujia Tank	Xicang Bridge	Cao Bridge
Fuzzy matter-element model	V	VI	VI	VI	VI
Comprehensive index method	V	VI	VI	VI	VI
Fuzzy comprehensive method	V	III	III	III	III
Bayesian method	V	III	III	III	III
Improved fuzzy matter-element model	V	VI	VI	VI	VI

**Table 5:** Membership degree of the fuzzy comprehensive assessment along the Caoqiao River.

Station	I	II	III	IV	V	Level
Zhongxi Bridge	0.00	0.00	0.28	0.14	0.59	V
Xujia Tank	0.00	0.09	0.42	0.27	0.22	III
Zhakou	0.00	0.03	0.36	0.29	0.32	III
Cao Bridge	0.00	0.00	0.47	0.25	0.23	III
Xicang Bridge	0.00	0.11	0.48	0.20	0.22	III

**Table 6:** Membership degree of the Bayesian method in five stations along the Caoqiao River.

Station	I	II	III	IV	V	Level
Zhongxi Bridge	0.09	0.10	0.25	0.15	0.41	V
Xujia tank	0.11	0.14	0.36	0.27	0.12	III
Zhakou	0.08	0.11	0.35	0.29	0.17	III
Cao Bridge	0.10	0.17	0.36	0.27	0.10	III
Xicang Bridge	0.12	0.15	0.33	0.16	0.24	III
